# Fetal Alcohol Syndrome Among Children Aged 7–9 Years — Arizona, Colorado, and New York, 2010

**Published:** 2015-01-30

**Authors:** Deborah J. Fox, Sydney Pettygrove, Christopher Cunniff, Leslie A. O’Leary, Suzanne M. Gilboa, Jacquelyn Bertrand, Charlotte M. Druschel, April Breen, Luther Robinson, Linnette Ortiz, Jaime L. Frías, Margaret Ruttenber, Donald Klumb, F. John Meaney

**Affiliations:** 1 Bureau of Environmental and Occupational Epidemiology, New York State Department of Health; 2 College of Public Health, University of Arizona; 3 Department of Pediatrics, University of Arizona; 4 Division of Birth Defects and Developmental Disabilities, National Center on Birth Defects and Developmental Disabilities, CDC; 5 Western Regional Office, New York State Department of Health; 6 Women and Children’s Hospital of Buffalo, Buffalo, New York; 7 McKing Consulting Corporation, Fairfax, Virginia; 8 Colorado Responds to Children with Special Needs, Colorado Department of Public Health and Environment; 9 Sewall Child Developmental Center, Denver, Colorado

Fetal alcohol syndrome (FAS) is a serious birth defect and developmental disorder caused by in utero exposure to alcohol (1). Assessment of the public health burden of FAS through surveillance has proven difficult; there is wide variation in reported prevalence depending on the study population and surveillance method. Generally, records-based birth prevalence studies report estimates of 0.2–1.5 per 1,000 live births (2), whereas studies that use in-person, expert assessment of school-aged children in a community report estimates of 6–9 per 1,000 population (3). The Fetal Alcohol Syndrome Surveillance Network II addressed some of the challenges in records-based ascertainment by assessing a period prevalence of FAS among children aged 7–9 years in Arizona, Colorado, and New York (4). The prevalence across sites ranged from 0.3 to 0.8 per 1,000 children. Prevalence of FAS was highest among American Indian/Alaska Native children and lowest among Hispanic children. These estimates continue to be much lower than those obtained from studies using in-person, expert assessment. Factors that might contribute to this discrepancy include 1) inadequate recognition of the physical and behavioral characteristics of FAS by clinical care providers; 2) insufficient documentation of those characteristics in the medical record; and 3) failure to consider prenatal alcohol exposure with diagnoses of behavioral and learning problems. Addressing these factors through training of medical and allied health providers can lead to practice changes, ultimately increasing recognition and documentation of the characteristics of FAS.

In 2009, CDC funded three sites, Arizona (statewide), Colorado (Denver-Boulder Consolidated Metropolitan Statistical Area), and New York (nine western counties), to conduct population-based surveillance of FAS in children aged 7–9 years who resided within the catchment areas in 2010. The surveillance methodology used by the sites is described in detail elsewhere (4). Sites used the standardized, multiple-source methodology developed by the Fetal Alcohol Syndrome Surveillance Network (2) that relied on passive reporting and active review of records from various sources to identify children with suspected FAS. Data from sources such as genetic and developmental clinics, hospital discharge files, Medicaid claims, health maintenance organization records, and the juvenile justice system were used for case finding.

A surveillance case definition (Table 1) was developed based on the 1996 Institute of Medicine report on FAS (1) and refined to reflect the older ages of the children in this cohort. Documentation of the features characteristic of FAS formed the basis of the case definition: facial dysmorphology, central nervous system (CNS) abnormalities, and growth deficiency. Maternal alcohol use during pregnancy was abstracted when available, but because of difficulty in obtaining reliable and valid documentation of this information, it was not required to meet the surveillance case definition. A confirmed case of FAS had documentation of facial features, CNS abnormalities, and growth deficiency; a probable case of FAS had documentation of facial features and either CNS abnormalities or growth deficiency (Table 1). Confirmed and probable cases were combined to estimate the prevalence of FAS. The denominator was the total number of children aged 7–9 years who resided in the catchment areas based on 2010 census estimates (5). Child’s race/ethnicity was reported if available; if the child’s race/ethnicity was missing, the race/ethnicity of the birth mother was used. Hispanic ethnicity was given priority over race, consistent with CDC’s National Center for Health Statistics guidelines.

The overall prevalence of FAS was 0.3 (95% confidence interval [CI] = 0.3–0.4) per 1,000 children aged 7–9 years; the site specific prevalence was 0.3 (CI = 0.2–0.3) in Arizona, 0.3 (CI = 0.2–0.4) in Colorado, and 0.8 (CI = 0.6–1.0) in New York (Table 2). Prevalence of FAS was highest among American Indian/Alaska Native children (2.0 [CI = 1.4–2.8] per 1,000 children aged 7–9 years) and lowest among Hispanic children (0.2 [CI = 0.1–0.2]). There were no differences in the prevalence of FAS by child’s age or sex.

## Discussion

Despite the older age cohort and focus on a period prevalence, the prevalence estimates obtained from the Fetal Alcohol Syndrome Surveillance Network II are similar to previously reported birth prevalence estimates using records-based methodology and much lower than those estimated by in-person, expert assessment of children (3). Factors that might contribute to this discrepancy include 1) inadequate recognition of the physical and behavioral characteristics of FAS by clinical care providers; 2) insufficient documentation of those characteristics in the medical record and; 3) failure to consider prenatal alcohol exposure with diagnoses of behavioral and learning problems.

That these factors might contribute to the discrepancy is supported by the findings of a survey of pediatricians published in 2006 in which more than two-thirds of respondents reported a lack of training as the primary reason for not making a FAS diagnosis (6). More than half of respondents indicated that they had no formal training on the recognition, diagnosis, or treatment of FAS, and two-thirds thought this diagnosis would stigmatize the family and child (6). The lack of training has a cascading effect: clinicians do not recognize and document physical and behavioral characteristics that might lead to a more complete clinical evaluation or that would serve as a trigger for a records-based surveillance system to identify the child as potentially having FAS. Further, maternal prenatal records are not routinely linked to a child’s birth or neonatal record at the hospital, meaning that prenatal alcohol exposure, if documented in the maternal record, is not known to pediatric clinicians when interpreting physical or behavioral characteristics of the child. Finally, some clinicians are hesitant to consider possible prenatal alcohol exposure in the diagnosis of behavioral and learning problems because services or interventions specific to FAS are not available in their community or clinicians are unaware of such services in their community (6)*.*

In 2014, CDC funded six Fetal Alcohol Spectrum Disorders (FASD) Practice and Implementation Centers.* These centers are designed to promote practice change among providers in the areas of FASD prevention, identification, and treatment. Two of the six centers will focus on pediatricians and are partnering with the American Academy of Pediatrics. Focused development of practice guidelines for pediatric clinicians through these Practice and Implementation Centers along with the broad-based dissemination capabilities of the American Academy of Pediatrics can improve identification, documentation, and clinical management of children with FAS, thereby strengthening the infrastructure needed for FAS records-based surveillance.

Collection of accurate population-based surveillance data for FAS is an important public health activity. In addition to providing an estimate of the public health burden of FAS, these data provide critical information to those planning clinical, behavioral, and educational interventions to support children with FAS and their families. Such services have been shown to reduce the risk for secondary conditions in this vulnerable population (7). Because many communities plan for service provision based on the prevalence estimates from records-based systems, the need for FAS specific treatments, interventions, and services might not be recognized.

What is already known on this topic?

Fetal alcohol syndrome (FAS) is a serious birth defect and developmental disorder caused by in utero exposure to alcohol. Its reported prevalence varies widely, reflecting differences in study populations and surveillance methods.

What is added by this report?

The prevalence of FAS in children aged 7–9 years in 2010 was 0.3 per 1,000 children in Arizona, 0.3 in Colorado, and 0.8 in New York, with a pooled prevalence of 0.3. These estimates are consistent with previous records-based surveillance estimates but substantially lower than estimates obtained from in-person, expert assessment of school-aged children in the community.

What are the implications for public health practice?

The lower estimates from records-based surveillance might be attributable to the following factors: 1) inadequate recognition of the physical and behavioral characteristics of FAS by clinical care providers; 2) insufficient documentation of those characteristics in the medical record; and 3) failure to consider prenatal alcohol exposure with diagnoses of behavioral and learning problems. Addressing these factors through training of medical and allied health providers can lead to practice changes, ultimately increasing recognition and documentation of the characteristics of FAS.

Surveillance of FAS also provides the opportunity to measure the effectiveness of public health interventions aimed at reducing the number of children at risk for FAS because of in utero alcohol exposure. Alcohol consumption during pregnancy is common. During 2006–2010, 7.6% of pregnant women reported drinking alcohol, with 1.4% reporting binge drinking (8)*.* Further, over 50% of pregnancies are unplanned (9), and alcohol exposure can harm the fetus even before the pregnancy is recognized (1)*.* FAS surveillance could provide evidence of the effectiveness of approaches to reduce alcohol consumption during pregnancy. One primary prevention strategy is alcohol screening and brief intervention. A California study found that pregnant women who received alcohol screening and brief intervention at a social service agency were five times more likely to abstain from alcohol during the remainder of their pregnancy and delivered infants who were healthier on several newborn measures (10)*.*

Recognition of children with FAS is critically important to ensure their access to appropriate services and interventions. However, identifying affected children through population-based surveillance continues to be a challenge. Prevalence estimates from the Fetal Alcohol Syndrome Surveillance Network II demonstrate that FAS is still underrecognized. Efforts that address the factors that contribute to this underrecognition might lead to practice changes, ultimately increasing recognition and documentation of the physical and behavioral characteristics of FAS. With increased recognition and documentation, records-based surveillance of FAS might yield estimates more similar to those based on in-person, expert assessment of school-aged children in a community.

## Figures and Tables

**TABLE 1:** Fetal alcohol syndrome (FAS) surveillance case definition* — Fetal Alcohol Syndrome Surveillance Network II, 2009–2014

Diagnostic category	Phenotype positive		

	Face	Central nervous system (CNS)	Growth
Confirmed FAS phenotype with or without documentation† of in utero alcohol exposure	Abnormal facial features consistent with FAS as reported by a physician or Two of the following: short palpebral fissures abnormal philtrum thin upper lip	At least one structural or functional anomaly **Structural** Head circumference ≤10th percentile at birth or any age or **Functional** Standardized measure of functioning in at least two of nine domains ≥1 standard deviations below the mean or diagnosis of developmental delay by a qualified examiner or Standardized measure of IQ ≥2 standard deviations below the mean on a standardized test or diagnosis of intellectual disability by a qualified examiner or ADD or ADHD diagnosed by a qualified evaluator	Growth delay indicated in at least one of the following: **Intrauterine** Weight or height corrected for gestational age ≤10th percentile or **Postnatal** Weight or height ≤10th percentile for age or Weight for height ≤10th percentile
Probable FAS phenotype with or without documentation† of in utero alcohol exposure	Same as confirmed	Must meet either CNS or growth criteria as outlined in the confirmed phenotype	
Suspected	All children referred into the surveillance system.		

**Abbreviations:** IQ = intelligence quotient; ADD = attention deficit disorder; ADHD = attention deficit hyperactivity disorder.

* Operationalized from the recommendations of the Institute of Medicine (Fetal alcohol syndrome: diagnosis, epidemiology, prevention, and treatment. Washington, DC: National Academy Press; 1996).

† Documentation in any abstracted record of maternal alcohol use during the index pregnancy.

**TABLE 2:** Prevalence (per 1,000) of fetal alcohol syndrome among children aged 7–9 years, by sex, race/ethnicity, and age — Arizona, Colorado, and New York,* 2010

Characteristic	Arizona			Colorado			New York			Total		
			
	Population	No. of cases	Prevalence (95% CI)	Population	No. of cases	Prevalence (95% CI)	Population	No. of cases	Prevalence (95% CI)	Population	No. of cases	Prevalence (95% CI)
**Total**	**271,895**	**67**	**0.3 (0.2–0.3)**	**117,638**	**29**	**0.3 (0.2–0.4)**	**82,924**	**65**	**0.8 (0.6–1.0)**	**472,457**	**161**	**0.3 (0.3–0.4)**
**Sex**												
Male	138,469	36	0.3 (0.2–0.4)	60,008	15	0.3 (0.1–0.4)	42,292	35	0.8 (0.6–1.1)	**240,769**	**86**	**0.4 (0.3–0.4)**
Female	133,426	31	0.2 (0.2–0.3)	57,630	14	0.2 (0.1–0.4)	40,632	30	0.7 (0.5–1.0)	**231,688**	**75**	**0.3 (0.3–0.4)**
**Race/Ethnicity**												
White, non-Hispanic	112,784	14	0.1 (0.1–0.2)	62,672	17	0.3 (0.2–0.4)	57,753	29	0.5 (0.3–0.7)	**233,209**	**60**	**0.3 (0.2–0.3)**
Black, non-Hispanic	10,756	4	0.4 (0.1–0.9)	6,197	3	0.5 (0.1–1.3)	12,014	22	1.8 (1.2–2.7)	**28,967**	**29**	**1.0 (0.7–1.4)**
AI/AN, non-Hispanic	12,956	25	1.9 (1.3–2.8)	458	1	2.2 (0.1–9.6)	524	2	3.8 (0.6–11.7)	**13,938**	**28**	**2.0 (1.4–2.8)**
A/PI, multiple, or other, non-Hispanic	16,607	3	0.2 (0.1–0.5)	9,694	0		5,478	2	0.4 (0.1–1.1)	**31,779**	**5**	**0.2 (0.1–0.3)**
Hispanic	118,792	12	0.1 (0.1–0.2)	38,617	7	0.2 (0.1–0.4)	7,155	6	0.8 (0.3–1.7)	**164,564**	**25**	**0.2 (0.1–0.2)**
Missing		9			1			4			**14**	
**Age (yrs)**												
7	90,407	26	0.3 (0.2–0.4)	39,795	10	0.3 (0.1–0.4)	27,225	13	0.5 (0.3–0.8)	**157,427**	**49**	**0.3 (0.2–0.4)**
8	89,191	21	0.2 (0.2–0.4)	38,806	11	0.3 (0.2–0.5)	27,519	26	0.9 (0.6–1.4)	**155,516**	**58**	**0.4 (0.3–0.5)**
9	92,297	20	0.2 (0.1–0.3)	39,037	8	0.2 (0.1–0.4)	28,180	26	0.9 (0.6–1.3)	**159,514**	**54**	**0.3 (0.3–0.4)**

**Abbreviations:** CI = confidence interval; AI/AN = American Indian/Alaska Native; A/PI = Asian/Pacific Islander.

* Surveillance areas: Arizona, statewide; Colorado, Denver-Boulder Consolidated Metropolitan Statistical Area; New York, nine western counties.

## Footnotes

* Additional information about this initiative available at http://www.cdc.gov/ncbddd/fasd/training.html.
